# Mitochondrial genome variation of mosquito species in the subgenus Stegomyia of the genus Aedes (Diptera: Culicidae)

**DOI:** 10.18699/vjgb-25-25

**Published:** 2025-04

**Authors:** A.G. Bega, I.I. Goryacheva, A.V. Moskaev, B.V. Andrianov

**Affiliations:** Federal State University of Education, Mytishchi, Moscow Region, Russia Vavilov Institute of General Genetics of the Russian Academy of Sciences, Moscow, Russia; Federal State University of Education, Mytishchi, Moscow Region, Russia Vavilov Institute of General Genetics of the Russian Academy of Sciences, Moscow, Russia; Federal State University of Education, Mytishchi, Moscow Region, Russia Vernadsky Russian State University of National Economy, Balashikha, Moscow Region, Russia; Vavilov Institute of General Genetics of the Russian Academy of Sciences, Moscow, Russia

**Keywords:** invasive species, mitochondrial genome, phylogenetic analysis, mtDNAFor, инвазионный вид, митохондриальный геном, филогенетический анализ, мтДНК

## Abstract

Mosquitoes in the subgenus Stegomyia of the genus Aedes are vectors of a number of vertebrate viruses, including human arboviral fevers. Of particular interest is the study of the genetic characteristics of invasive populations of species in this group. We obtained, annotated and described the mitochondrial genomes of three Stegomyia mosquito species of the genus Aedes: Ae. albopictus, Ae. flavopictus and Ae. sibiricus. The mitochondrial genomes of Ae. flavopictus and Ae. sibiricus were obtained from mosquitoes from synanthropic populations in the Russian Far East. The mitochondrial genome of Ae. sibiricus is presented for the first time. The mitochondrial genome of Ae. albopictus was obtained for the C6/36 cell line. We selected three primer sets, for each mosquito species, that amplify the entire mitochondrial genome except for the control region and sequenced the genomes using the Sanger method. All three new genomes have an identical gene order. We identified 13 canonical protein-coding genes, 2 ribosomal RNA genes, and 22 transport RNA genes. Protein-coding genes have canonical start and stop codons with two exceptions. The canonical stop codon “TAA” is incomplete in the cox1 and cox2 genes. The cox1 gene lacks the canonical start codon for methionine. Nucleotide variability is mainly represented by point nucleotide substitutions. A phylogenetic analysis of the nucleotide sequences of complete mitochondrial genomes of all known mosquitoes species in the subgenus Stegomyia of the genus Aedes was performed. The data obtained made it possible to measure the ratio of synonymous to non-synonymous substitutions (Ka/Ks) in specific protein-coding genes.

## Abstract (ru)

Комары рода Aedes, подрода Stegomyia являются переносчиками ряда вирусов позвоночных, в том числе возбудителей арбовирусных лихорадок человека. Особый интерес представляет изучение генетических особенностей синантропных популяций видов этой группы. Мы получили, аннотировали и описали митохондриальные геномы трех видов комаров рода Aedes, подрода Stegomyia: Ae. albopictus, Ae. flavopictus и Ae. sibiricus. Митохондриальные геномы Ae. flavopictus и Ae. sibiricus были получены от комаров из синантропных популяций с Дальнего Востока России. Митохондриальный геном Ae. sibiricus представлен нами впервые Митохондриальный геном Ae. albopictus был получен для клеточной линии C6/36. Мы подобрали три комплекта праймеров для каждого из видов комаров, которые амплифицируют весь митохондриальный геном, кроме контрольной области, и отсеквенировали геномы методом Сэнгера. Все три новых генома имеют одинаковый порядок расположения генов. Идентифицировано 13 канонических белок-кодирующих генов, 2 гена рибосомальной РНК, 22 гена транспортной РНК. Белок-кодирующие гены имеют канонические старт- и стоп-кодоны за двумя исключениями. Канонический стоп-кодон «TAA» неполный в генах cox1 и cox2. В гене cox1 отсутствует канонический старт-кодон для метионина. Нуклеотидная изменчивость представлена в основном точковыми нуклеотидными замещениями. Инсерции-делеции имеются в областях межгенных спейсеров. Проведен филогенетический анализ нуклеотидных последовательностей полных митохондриальных геномов всех известных видов комаров рода Aedes, подрода Stegomyia. Полученные данные позволили провести измерение соотношения синонимичных и несинонимичных замен (Ka/Ks) в конкретных белок-кодирующих генах.

## Introduction

Mosquitoes of the genus Aedes, subgenus Stegomyia, are the main vectors of dengue, yellow fever and other arbovirus infections worldwide (Weetman et al., 2018). Of greatest interest and practical importance are the invasive mosquito species in this group that form dense synanthropic populations. Aedes albopictusis Skuse, 1894 is an invasive species native to Southeast Asia that has spread to all continents except Antarctica in the last 50 years (Medlock et al., 2012). In the Russian Federation, this species is found in the southern European part of the country. The study of its genetic variability in Russia is mainly based on the analysis of the barcode fragment of the mitochondrial gene cox1 (Fedorova et al., 2019; Bega et al., 2022).

The subgenus Stegomyia is represented in Russia by three other species of mosquitoes found in the Far East and Siberia – Aedes flavopictus Yamada, 1921, Aedes sibiricus Danilov & Filippova, 1978 and Aedes galloisi Yamada, 1921. These three species are considered native forest species. Comparative analyses of the genetic structure of Ae. albopictus and Ae. flavopictus populations from the Korean peninsula support this hypothesis (Shin, Jung, 2021). Previously, Ae. flavopictus and Ae. sibiricus did not form dense populations in the Far East and were only found as isolated specimens (Gutsevich et al., 1970). Recently there have been reports of sightings of these species in urban areas (Berlov, Kuberskaya, 2021; Berlov et al., 2021). We have obtained data on range expansion and the formation of dense synanthropic populations of Ae. flavopictus and Ae. sibiricus in the Russian Far East (Bega et al., 2022). This probably indicates the beginning of the formation of invasive populations of these species.

In this paper, we present the results of sequencing the mitochondrial genomes of representatives of potentially invasive populations of Ae. flavopictus and Ae. sibiricus, and the mitochondrial genome of the cell line Ae. albopictus C6/36, as well as the phylogenetic analysis of the obtained sequences

The mitochondrial genome of Ae. albopictus is now well characterised, but some points remain controversial. The mitochondrial genomes of mosquitoes from the island of Taiwan, including the reference genome (ID NC_006817), have reading frame shifts and abnormal stop codons. This may be due to the fact that the sample was taken from an insular and presumably indigenous population. It may also be a consequence of the inclusion of nuclear copies of mitochondrial genes, or Numts, in the mitochondrial genome. Some sequences of the mitochondrial genome of Ae. albopictus represented in GenBank have deletions and poly(A) spacers (Battaglia et al., 2016; Ze-Ze et al., 2020). The features of the mitochondrial genome of Ae. albopictus cell culture have not been previously studied. C6/36 culture was obtained from mosquitoes, the place of capture of which is not precisely known (Singh, 1967). To date, the culture has been passaged in the laboratory for more than 50 years. Under cell culture conditions, with constant temperature and nutrient levels, the cells do not experience the selection factors that natural mosquito populations do. Obtaining the mitochondrial genome of a C6/36 cell culture is of interest because it shows which mitochondrial genes are under selection in natural populations. At the time of publication, only two mitochondrial genome sequences of Ae. flavopictus, NC_050044 and MT501510, from the southern part of the species range were available in NCBI GenBank. The genome we obtained represents a previously uncharacterised northern part of the range. The mitochondrial genome of Ae. sibiricus was obtained for the first time in this study. The NCBI GenBank had a mitochondrial genome for the closely related species Ae. galloisi. The sequences obtained in this study are of interest and can be used for further studies on the genetic characteristics of mosquitoes of the subgenus Stegomyia.

## Materials and methods

Specimen collection and species identification. Mosquito samples were collected in the Russian Far East in the summer of 2020. We trapped Ae. flavopictus in Khabarovsk and Ae. sibiricus in Svobodny city, Amur region. Aedes albopictus clone C6/36 is a commercially available mosquito cell line isolated from larvae of this species (Singh, 1967). Species identification by morphological characters was carried out according to the keys in the identifiers (Gutsevich et al., 1970; Tanaka, 1979; Ree, 2003).

The taxonomic status of the mosquito we defined as Ae. sibiricus should be mentioned separately. Not all of the identifiers mentioned above include data on the separation of the species Ae. sibiricus from the previously described Ae. galloisi (Danilov, Filippova, 1978). We used keys to identify these species based on the colour of the legs and the structure of the hypopygium in males (Danilov, Filippova, 1978; Poltoratskaya, Mirzaeva, 2013). The species Ae. sibiricus is currently listed in the Mosquitoes of the World catalogue of blood-sucking mosquitoes (Wilkerson et al., 2021); however, the description of the species is only published in Russian and therefore Ae. sibiricus is not included in the GenBank taxonomic database.

DNA isolation and sequencing of the mitochondrial genome. Total DNA was isolated from individual adult mosquitoes. Each individual was homogenised in lysis solution. The composition of the lysis solution was as follows: 500 mM Tris-EDTA pH = 8.0, 100 μg/ml Proteinase K, 1 % Sodium N-lauroylsarcosinate, 100 mM NaCl. Lysis was performed at 50 °C for 3 hours. After lysis, the DNA was extracted with phenol. The phenol was in the upper layer. Two volumes of water were added to the resulting DNA solution, then the DNA was precipitated with isopropyl alcohol. After purification, the DNA was dissolved in deionised water.

Mitochondrial genomes were amplified using the Encyclo Plus PCR kit (Evrogen, Russia) and sequenced using the Sanger method. We selected the primers ourselves using Primer3 software (Rozen, Skaletsky, 2000) based on the Ae. albopictus mitochondrial genome published in the paper (Battaglia et al., 2016). PCR amplification for all primer pairs we selected was performed at an annealing temperature of 58 °C. The list of primers used is shown in Tables 1–3.

**Table 1.**
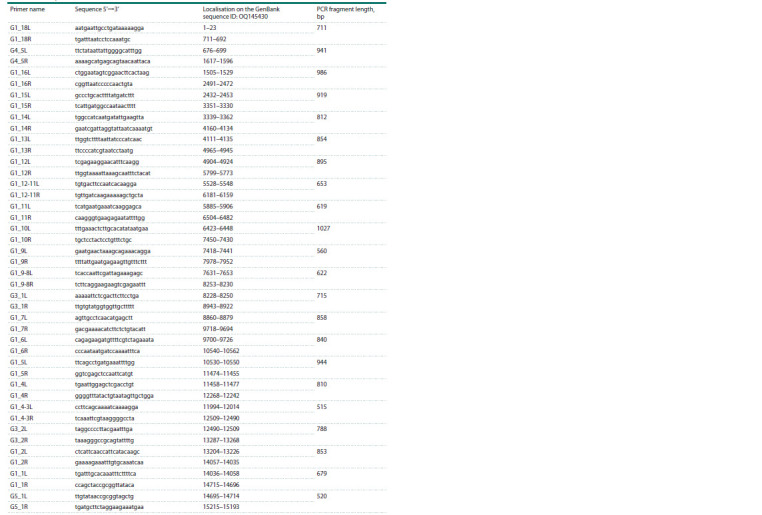
List of primers used to obtain the nucleotide sequence of the complete mitochondrial genome of C6/36 Ae. albopictus cell culture

**Table 2.**
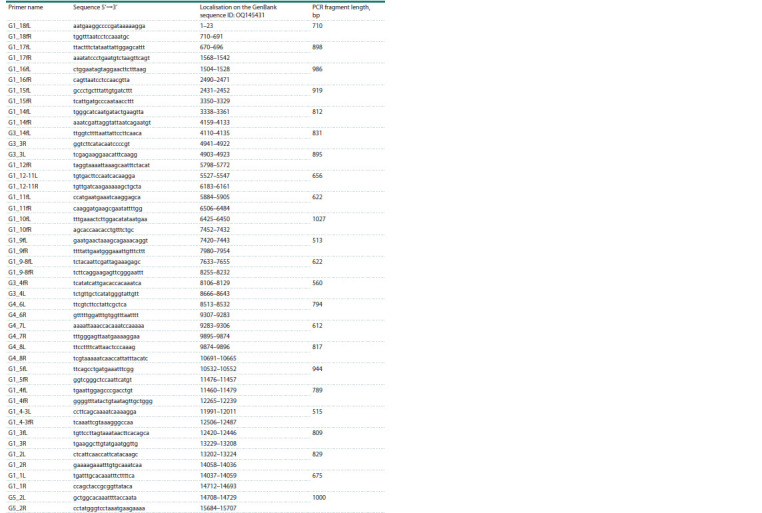
List of primers used to obtain the nucleotide sequence of the complete mitochondrial genome of Ae. flavopictus

**Table 3.**
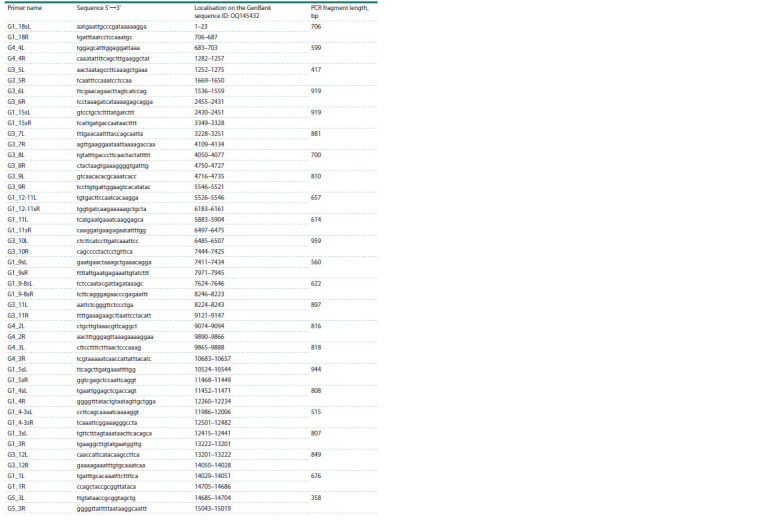
List of primers used to obtain the nucleotide sequence of the complete mitochondrial genome of Ae. sibiricus

Bioinformatics analysis. Sequences were analysed using BLAST software to identify mitochondrial genes. Open reading frame start and stop codons were determined by comparison with start and stop codons of orthologous protein-coding genes in GenBank. Phylogenetic analysis was performed using the MEGA7 programme (Kumar et al., 2016). Sequences obtained from sequencing were aligned to sequences in the databases using NCBI resources (http://www.ncbi.nlm.nih.gov). We used the multiple sequence alignment algorithm Clustal W (Thompson et al., 1994). Visualisation of the mitochondrial genome ring was performed using Chloroplot software (Zheng et al., 2020). The algorithm for calculating the Ka/Ks ratio is described in the paper (Wang D. et al., 2011). We have carried out the calculation using the KaKs_Calculator software (Zhang Z. et al., 2006) using a simple substitution correction method (NG) (Nei, Gojobori, 1986). Suppose the length of the DNA sequence being compared is n and the number of substitutions between the sequences being compared is m. To calculate Ka and Ks, we need to count the number of synonymous (S) and non-synonymous (N) sites (S + N = n) and the number of synonymous (Sd) and non-synonymous (Nd) substitutions (Sd + Nd = m). Then, after correction for multiple substitutions, (Nd/N) and (Sd/S) can represent Ka and Ks, respectively. This is because the observed number of substitutions underestimates the true number of substitutions due to the divergence of sequences over time. Therefore, the calculation involved three steps: counting S and N, counting Sd and Nd, and correcting for multiple substitutions. Link to the programme distribution https://ngdc.cncb.ac.cn/ biocode/tools/BT000001.

## Results

Organisation of the derived mitochondrial genomes

The mitochondrial genomes of three mosquito species of the genus Aedes, subgenus Stegomyia (Ae. albopictus, Ae. flavopictus and Ae. sibiricus) are identical in gene order and similar in nucleotide sequence. The length of the mitochondrial genome excluding the length of control regions is as follows: Ae. albopictus 14,900 bp, Ae. flavopictus 14,893 bp, Ae. sibiricus 14,886 bp. Nucleotide variability is represented by point nucleotide substitutions. When comparing the mitochondrial genomes of Ae. albopictus and Ae. flavopictus, the degree of nucleotide divergence is minimal (5.74 %). The maximum degree of nucleotide divergence is observed when comparing the nucleotide sequences of the mitochondrial genomes of Ae. albopictus and Ae. sibiricus (7.51 %). The mitochondrial genomes of Ae. flavopictus and Ae. sibiricus differ by 6.62 %. All three mitochondrial genomes have a strong A + T = 78.4 % bias, which is characteristic of Diptera mitochondrial genomes

**Fig. 1.**
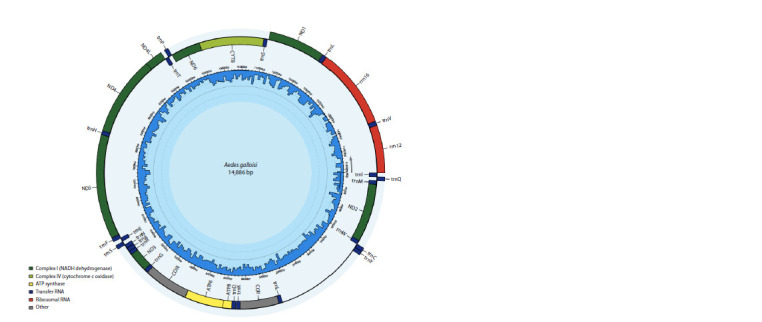
Mitochondrial genome of Ae. sibiricus without the control site located between 12S rRNA and tRNA-Ile. The nucleotide sequence has been deposited in the GenBank database under accession number OQ145432. The genome is registered as Ae. galloisi because the separation of the closely related species Ae. galloisi and Ae. sibiricus is not yet generally accepted, and the species Ae. sibiricus is not yet represented in the GenBank systematic database. We believe that the correct species name for the collected mosquitoes is Ae. sibiricus.

We identified 13 canonical protein-coding genes (PCGs), 2 ribosomal RNA genes, and 22 transport RNA genes. All PCGs have canonical start and stop codons with two exceptions. The canonical stop codon “TAA” is incomplete in the cox1 and cox2 genes. It is thought that the missing base “A” is added during RNA processing. In addition, the canonical start codon for methionine is missing in the cox1 gene. The heavy (J) strand contains 22 genes, including 9 PCGs and 13 tRNA. The remaining 15 genes are encoded on the light strand (N-strand), including 4 PCGs, 2 rRNA and 9 tRNA. The ring genetic map of the mitochondrial genome of Ae. sibiricus is shown in Figure 1.

Phylogenetic analysis

**Fig. 2.**
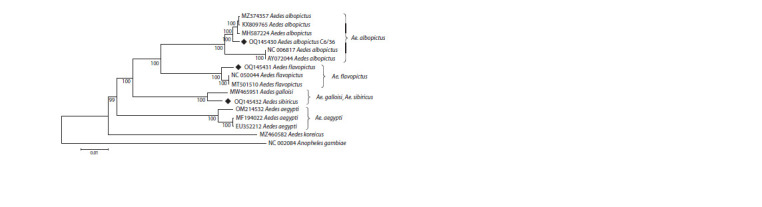
NJ dendrogram of complete mitochondrial genomes. The dendrogram is constructed using the maximum likelihood method. Branch lengths are expressed as the number of base substitutions per site. Bootstrap support values are shown next to the nodes (10,000 replicates). The complete mitochondrial genomes of Ae. koreicus and Anopheles gambiae were used as an external group. The mitochondrial genomes obtained in this study are marked with a diamond in the figure. The nucleotide sequences are registered in the GenBank database under the numbers OQ145430–OQ145432.

The phylogenetic analysis of the nucleotide sequences of the mosquito mitochondrial genomes we obtained, using all available sequences of the mitochondrial genomes of Ae. albopictus, Ae. flavopictus, Ae. aegypti and Ae. galloisi registered in GenBank, is shown in Figure 2. The comparison region included the entire mitochondrial genome except for the control region

Ae. albopictus, Ae. aegypti and Ae. flavopictus form independent clusters with high bootstrap support values. The mitochondrial genome of the C6/36 cell line clusters with the mitochondrial genomes of Ae. albopictus from the invasive part of the species’ range. Ae. sibiricus and Ae. galloisi are clustered together

PCGs variability analysis

**Fig. 3.**
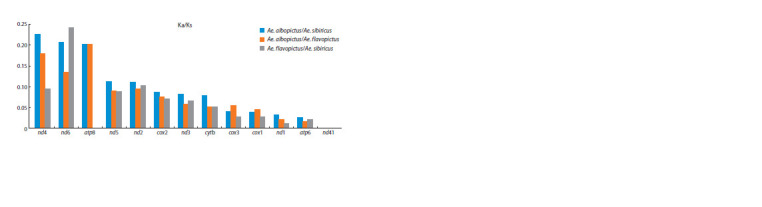
Pairwise interspecies comparisons of the Ka/Ks ratio in protein-coding mitochondrial genes

We calculated the frequency ratio of point nucleotide substitutions leading to a change in the amino acid sequence (nonsynonymous substitutions, Ka) or not leading to a change in the amino acid sequence of the protein (synonymous substitutions, Ks) Ka/Ks for PCGs in a pairwise comparison of the mitochondrial genomes obtained in this study: Ae. albopictus and Ae. sibiricus, Ae. albopictus and Ae. flavopictus, Ae. flavopictus and Ae. sibiricus (Fig. 3). The mosquito species we studied are closely related, their habitats overlap slightly, but the centres of their ranges belong to different natural and climatic zones. Ae. albopictus is mainly restricted to tropical and subtropical climates, while Ae. flavopictus and Ae. sibiricus are restricted to temperate climates. At the same time, Ae. flavopictus predominates in zones with a monsoon climate, and Ae. sibiricus, in zones with a strongly continental climate. The pairwise comparison of PCGs was used to identify differences that may be adaptively relevant between the mosquito species. In Figure 3, Ka/Ks values are ranked in descending order based on the comparison of Ae. albopictus and Ae. sibiricus

Ka/Ks ratios do not exceed 0.25 in all pairwise comparisons, indicating strong stabilising selection (Yang, Bielawski, 2000; Guo et al., 2021; Xing et al., 2022). The most variable genes between Ae. albopictus/Ae. sibiricus and Ae. albopictus/ Ae. flavopictus are nd4, nd6 and atp8. The most conserved genes are nd1, atp6, nd4l and cox1

**Fig. 4.**
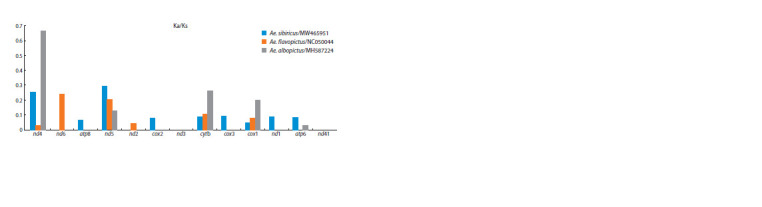
Pairwise intraspecific comparisons of nucleotide variability in the magnitude of the Ka/Ks ratio of mitochondrial proteincoding genes

In addition to interspecific comparisons, we performed intraspecific pairwise comparisons to assess the intraspecific variability of mitochondrial PCGs. The mitochondrial genome of Ae. albopictus cell culture C6/36 obtained in this study was compared with the genome of Ae. albopictus from China, GenBank ID MH587224. We compared the mitochondrial genome of Ae. flavopictus with the genome of Ae. flavopictus from Japan, GenBank ID NC050044, and the mitochondrial genome of Ae. sibiricus with the genome of Ae. galloisi from Japan, GenBank ID MW465951. The values of the Ka/Ks ratios are shown in Figure 4. The order of the genes is identical to the order of the gene rankings in Figure 3.

Within the Ae. flavopictus species, the highest Ka/Ks values were observed in the genes nd5, nd6, cox1, cytb. The genes atp8, cox2, nd3, coх3, nd1, atp6, nd4l were conservative. When comparing the mitochondrial genomes of Ae. albopictus mosquitoes from the natural population and from C6/36 cell culture (Singh, 1967), the highest Ka/Ks ratio was observed in the genes nd4, cytb, coх1, nd5. The most conserved genes were: nd6, atp8, nd2, cox2, nd3, cox3, nd1, nd4l. When comparing the mitochondrial PCGs of Ae. sibiricus and Ae. galloisi, the highest Ka/Ks values were observed in the genes nd4, nd5, atp8, cox2, cytb, cox3, nd1, coх1, atp6. The following genes were conserved: nd6, nd2, nd3, nd4l.

## Discussion

Organisation of the derived mitochondrial genomes

The nucleotide divergence values obtained in this study between the three closely related mosquito species are comparable and correspond to their geographical distribution in eastern Asia. Ae. albopictus is the most thermophilic species, characteristic of China and southern Asia. Ae. sibiricus is the most northerly. Ae. flavopictus occupies a middle position (Bega et al., 2022).

The use of molecular genetic markers to identify mosquito species is based on the use of a threshold of acceptable intraspecific variability of a given marker. This threshold is determined empirically for each marker and for each systematic group of insects (Zhang H.Z. et al., 2017). For example, for many insect groups, the threshold for intraspecific nucleotide variability of the BOLD fragment of the mitochondrial gene cox1 is 3 % (Hebert et al., 2003). Intraspecific variability of Anopheles hyrcanus s. l. mosquitoes in the Russian Far East ranged from 0.36 to 1.09 %, interspecific variability – from 2.34 to 4.50 % (Khrabrova et al., 2015). The average intraspecific variability of mosquitoes in China for the cox1 barcode fragment was 0.39 % (Wang G. et al., 2012). For complete mitochondrial genomes, much information has been accumulated, but there are no generally accepted quantitative generalisations

Phylogenetic analysis

The Ae. albopictus mitochondrial genomes published to date can be divided into two groups. The first group was found on the island of Taiwan (presumably the native range of the species). Genomes from the second group were found in mosquitoes from the invasive part of the species range (Battaglia et al., 2016). The mitochondrial genome of the Ae. albopictus C6/36 cell line clustered with genomes belonging to the second group. The clustering obtained by analysing complete mitochondrial genomes is similar to that obtained in previous studies for the BOLD fragment of the cox1 gene (Bega et al., 2022).

PCGs variability analysis

The selection pressure on protein-coding genes can be assessed by determining their Ka/Ks ratio. We made such a comparison at the interspecific level by comparing the genomes obtained in this study. The highest Ka/Ks values for all PCGs, except nd6, cox1, cox3, were observed in the Ae. albopictus/ Ae. sibiricus comparison. This result is in good agreement with the differences between species in terms of habitat ecology. The greater the differences in habitat between the species, the more significant the substitutions in the PCGs. The distribution of Ka/Ks values from higher to lower values within the PCGs was generally similar in all three pairwise comparisons, except for some peculiarities. For example, when comparing species from the same geographical area of the Russian Far East, Ae. flavopictus/Ae. sibiricus, the Ka/Ks ratio for the nd4 gene was significantly lower and no nucleotide substitutions were found at all for atp8. Calculating the frequency of substitutions normalised to one nucleotide, we can conclude that the atp8 gene in mosquitoes of the Stegomyia subgenus is characterised by a lower frequency of nucleotide substitutions than that in other protein-coding mitochondrial genes. A higher Ka/Ks ratio in the atp8 gene compared to other proteincoding mitochondrial genes was shown in a comparison of two Lepidoptera species of the genus Gynaephora living in different high mountain environments (Zhang B. et al., 2021), in parasitic wasp (Xing et al., 2022), and in mosquitoes of the Anopheles genus (Guo et al., 2021). This is probably due to the absence of strict constraints on the primary structure of the functional atp8 protein. In the nd1, atp6, nd4l and cox1 genes, the total frequency of nucleotide substitutions is comparable to that of other mitochondrial genes, but the Ka/Ks values are low, which confirms that these genes are under strong selective pressure.

In contrast to interspecific comparisons, the distribution of Ka/Ks between PCGs in intraspecific comparisons does not show clearly expressed general regularities, but characterises the specificity of variability accumulation for each species.

When comparing the mitochondrial genomes of Ae. flavopictus, the highest Ka/Ks values are observed for the nd5, nd6, cox1 and cytb genes. This is due to the lower pressure of purifying selection. The pattern of intraspecific variability of these genes is similar to that found in the interspecific comparisons shown in Figure 3.

It is interesting to compare the mitochondrial genomes of Ae. albopictus mosquitoes from the natural population and from C6/36 cell culture. The highest Ka/Ks ratio is observed in the genes nd4, cytb, cox1, nd5. The value of the Ka/Ks ratio in this case exceeds the values characteristic of both interspecific and intraspecific comparisons by a multitude, which allows us to conclude that selection in cell culture conditions is weak or absent. At the same time, the presence of fully conserved genes is observed: nd6, atp8, nd2, cox2, nd3, cox3, nd1, nd4l. This contrast in the variability of different genes of Ae. albopictus may be the result of the removal of a number of physiological constraints in cell culture conditions experienced by individuals in natural populations

The variability observed when comparing the mitochondrial genomes of Ae. sibiricus and MW465951 mosquitoes generally corresponds to the level of interspecific variability in Aedes mosquitoes of the Stegomyia subgenus, with the exception of two abnormal genes: nd6 and nd5. The normally highly variable nd6 gene is monomorphic in this comparison, which may be due to the presence of stabilising selection. The nd5 gene, on the other hand, contains an abnormally high number of non-synonymous substitutions.

## Conclusion

The study of the peculiarities of natural selection in invasive insect populations is still at the stage of accumulating material. One of the approaches used to detect the peculiarities of selection leading to the emergence of invasive populations in insects is to compare the mitochondrial genomes of native and invasive populations of the same species. Simultaneous coexistence of native and invasive populations is now known for many insect species, such as the Asian ladybird Harmonia axyrid (Brown et al., 2011), the Japanese grape leafhopper Arboridia kakogawana (Piccinno et al., 2024), and several others. The study of the mitochondrial genomes of species that successfully synanthropise and form dense populations in urbanised areas is of interest for the discovery of mitochondrial genes involved in the genetic control of the increased viability trait characteristic of invasive insect populations.

## Conflict of interest

The authors declare no conflict of interest.
